# Pregnancy in teenagers diagnosed with type 1 diabetes mellitus in childhood: a national population-based e-cohort study

**DOI:** 10.1007/s00125-019-05063-w

**Published:** 2019-12-20

**Authors:** Lowri A. Allen, Rebecca L. Cannings-John, Annette Evans, Daniel S. Thayer, Robert French, Shantini Paranjothy, David L. Fone, Colin M. Dayan, John W. Gregory

**Affiliations:** 1 Diabetes Research Group , C2 link corridoe University Hospital of Wales Heath Park, Cardiff, CF14 4XN UK; 2 South East Wales Trials Unit, Cardiff University, Cardiff, UK; 3 Division of Population Medicine, Cardiff University, Cardiff, UK; 4 SAIL Databank, School of Medicine, Swansea University, Swansea, UK

**Keywords:** Diabetes in childhood, Epidemiology, Pregnancy

## Abstract

**Aims/hypothesis:**

The aim of this study was to describe the characteristics and outcomes of pregnancies in a national cohort of teenage (<20 years) and young adult women (≥20 years) with and without childhood-onset (<15 years) type 1 diabetes. We hypothesised that, owing to poor glycaemic control during the teenage years, pregnancy outcomes would be poorer in teenage mothers with type 1 diabetes than young adult mothers with type 1 diabetes and mothers without diabetes.

**Methods:**

The Brecon Register of childhood-onset type 1 diabetes diagnosed in Wales since 1995 was linked to population-based datasets in the Secure Anonymised Information Linkage (SAIL) Databank, creating an electronic cohort (e-cohort) of legal births (live or stillbirths beyond 24 weeks’ gestation) to women aged less than 35 years between 1995 and 2013 in Wales. Teenage pregnancy rates were calculated based on the number of females in the same birth cohort in Wales. Pregnancy outcomes, including pre-eclampsia, preterm birth, low birthweight, macrosomia, congenital malformations, stillbirths and hospital admissions during the first year of life, were obtained from electronic records for the whole Welsh population. We used logistic and negative binomial regression to compare outcomes among teenage and young adult mothers with and without type 1 diabetes.

**Results:**

A total of 197,796 births were eligible for inclusion, including 330 to girls and women with childhood-onset type 1 diabetes, of whom 68 were teenagers (age 14–19 years, mean 17.9 years) and 262 were young adults (age 20–32 years, mean 24.0 years). The mean duration of diabetes was 14.3 years (9.7 years for teenagers; 15.5 years for young adults). Pregnancy rates were lower in teenagers with type 1 diabetes than in teenagers without diabetes (mean annual teenage pregnancy rate between 1999 and 2013: 8.6 vs 18.0 per 1000 teenage girls, respectively; *p* < 0.001). In the background population, teenage pregnancy was associated with deprivation (*p* < 0.001), but this was not the case for individuals with type 1 diabetes (*p* = 0.85). Glycaemic control was poor in teenage and young adult mothers with type 1 diabetes (mean HbA_1c_ based on closest value to conception: 81.3 and 80.2 mmol/mol [9.6% and 9.5%], respectively, *p* = 0.78). Glycaemic control improved during pregnancy in both groups but to a greater degree in young adults, who had significantly better glycaemic control than teenagers by the third trimester (mean HbA_1c_: 54.0 vs 67.4 mmol/mol [7.1% vs 8.3%], *p* = 0.01). All adverse outcomes were more common among mothers with type 1 diabetes than mothers without diabetes. Among those with type 1 diabetes, hospital admissions during the first year of life were more common among babies of teenage vs young adult mothers (adjusted OR 5.91 [95% CI 2.63, 13.25]). Other outcomes were no worse among teenage mothers with type 1 diabetes than among young adult mothers with diabetes.

**Conclusions/interpretation:**

Teenage girls with childhood-onset type 1 diabetes in Wales are less likely to have children than teenage girls without diabetes. Teenage pregnancy in girls with type 1 diabetes, unlike in the background population, is not associated with social deprivation. In our cohort, glycaemic control was poor in both teenage and young adult mothers with type 1 diabetes. Pregnancy outcomes were comparable between teenage and young adult mothers with type 1 diabetes, but hospital admissions during the first year of life were five times more common among babies of teenage mothers with type 1 diabetes than those of young adult mothers with diabetes.

**Electronic supplementary material:**

The online version of this article (10.1007/s00125-019-05063-w) contains peer-reviewed but unedited supplementary material, which is available to authorised users.

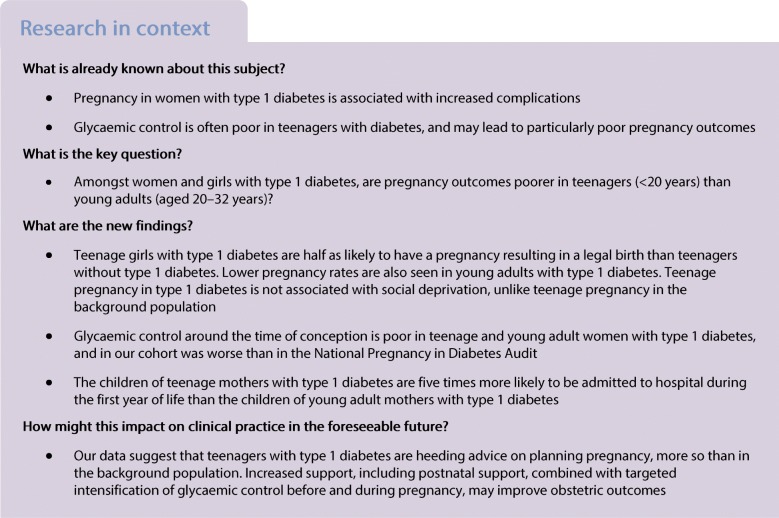



## Introduction

Pregnancy in type 1 diabetes is associated with increased complications, including pre-eclampsia, preterm birth, macrosomia, congenital malformations and stillbirths [1–7]. However, few studies have explored the relationship between maternal age and pregnancy outcomes in type 1 diabetes.

In the general population, pregnancies at the extremes of reproductive age are associated with increased complications [8–10]. Teenage pregnancy is associated with increased complications including low birthweight, premature birth and neonatal death [11, 12]. Contributing factors include high levels of deprivation and unplanned pregnancies, inadequate engagement with antenatal care and high smoking rates [11, 13]. Similar factors may adversely affect pregnancy in teenagers with type 1 diabetes. In addition, glycaemic control strongly influences pregnancy outcomes and is particularly poor in teenagers [14, 15]. Unplanned pregnancy may reduce the opportunities to optimise glycaemic control before conception. Pregnancy outcomes may therefore be particularly poor in teenagers with diabetes. Understanding the consequences of teenage pregnancy in diabetes is important as even small numbers of adverse outcomes, such as congenital malformations, can have major consequences.

The published literature on pregnancy outcomes in teenagers with type 1 diabetes is limited. A recently published study of pregnancy outcomes in teenagers with pregestational diabetes reported that adverse outcomes are more common in teenagers with pregestational diabetes than in teenagers without diabetes, but did not compare outcomes between teenagers with diabetes and older women with diabetes [16]. The largest published study to date comparing outcomes in teenagers and older women with type 1 diabetes included just 18 teenage pregnancies, and suggested that the children of teenagers with type 1 diabetes are at higher risk of congenital malformations [17]. Well-designed community-based studies are required to validate this, and to provide a comprehensive description of teenage pregnancies in type 1 diabetes and their outcomes.

The Brecon Group has a near-complete (98%) register of children with type 1 diabetes diagnosed prior to age 15 years in Wales since 1995 (*n* = 3289). This national community-based approach, along with minimal cross-border movement of participants, means it is representative of all individuals with childhood-onset type 1 diabetes in Wales. Linkage of the Brecon Register with other population-based datasets through the Secure Anonymised Information Linkage (SAIL) Databank has previously been used to demonstrate excess hospital admissions in children with type 1 diabetes [18]. Linkage with national datasets within SAIL Databank facilitates a cohort study of all pregnancies in Wales within a defined period, providing a more complete record of outcomes and avoiding the potential sources of bias observed in previous studies. In particular, pregnancy rates and outcomes can be compared with those of the background maternity population. The aim of this study was to use this approach to describe the characteristics of pregnancy in teenagers with and without childhood-onset type 1 diabetes, and to compare pregnancy outcomes between teenagers and young adult women. We hypothesised that outcomes would be poorest among teenage mothers with type 1 diabetes, due to poor glycaemic control and other adverse factors.

## Methods

### Study population

We included pregnancies resulting in legal births (live or stillbirths beyond 24 weeks’ gestation) in women under 35 years between 1995 and 2013 in Wales. The Brecon Register is a prospective cohort of newly diagnosed children with type 1 diabetes aged under 15 years in Wales since 1995, and contains individuals born from 1980 onwards.

We described the baseline characteristics of pregnancies in teenage (<20 years) and young adult women (≥20 years) with and without childhood-onset type 1 diabetes (diagnosed at <15 years), as identified from the Brecon cohort, and compared outcomes between groups. This definition of teenage pregnancy is that of the World Health Organization [19].

### Data linkage

Data linkage was undertaken within the SAIL Databank (Farr Institute@CIPHER at Swansea University) [20–22]. Individuals were assigned an anonymised linking field based on their National Health Service number, name, sex, date of birth and postcode. The anonymised linking field was used to link the datasets outlined below. Researchers did not have access to personal identifiable data.

The Wales Electronic Cohort for Children (WECC), which contains anonymised information on children born or living in Wales since 1990 [23], was used to define our cohort. Because detailed birth data are only available within the WECC for children born in Wales, births outside Wales were excluded. Data extracted included the week of birth, maternal age at birth, maternal socioeconomic status based on area of residence (Townsend deprivation quintiles), parity, maternal smoking (based on self-reporting to midwives), multiple births, delivery by Caesarean section, sex of baby, weeks’ gestation at delivery, birthweight, stillbirth, breastfeeding at 8 weeks, and neonatal and postneonatal deaths. Linkage to the Brecon Register identified maternal childhood-onset type 1 diabetes status.

The Congenital Anomaly Register and Information Service (CARIS) is a register of babies with congenital anomalies, with their mother resident in Wales at birth, since 1998 [24]. CARIS was used to identify children with congenital anomalies.

The Patient Episode Database for Wales (PEDW) was used to identify pre-eclampsia and NHS Wales hospital admissions during the first year of life [25].

Data from primary care are available for around 75% of general practices in Wales, with varying start dates, but most going back to at least 2000 [26]. We used these data to obtain HbA_1c_ values recorded for girls and women with diabetes from 1 year prior to conception to the delivery date.

### Pregnancy rates

The Brecon Register was used to establish the total number of teenage (<20 years) and young adult (20–35 years) women with childhood-onset type 1 diabetes in Wales during the study period. Combined birth records and census data from the Office for National Statistics were used to establish the total number of teenage and young adult women in the background population during the same period [27]. These data were used to calculate the proportion of teenage and young adult women, with and without diabetes, who had a pregnancy resulting in a legal birth.

### Outcomes

The study outcomes were maternal pre-eclampsia (as defined in the ICD, 10th revision [28]), preterm birth (birth before 37 weeks of pregnancy), macrosomia (birthweight ≥4000 g), low birthweight (≤2500 g), congenital malformations (as defined by the EUROCAT congenital anomalies registries [29]), stillbirths and hospital admissions during the first year of life.

Confounders of interest included maternal age, Townsend deprivation quintiles, parity, maternal smoking, delivery by Caesarean section, sex of the baby, gestation at delivery and breastfeeding at 8 weeks.

### Statistical analyses

Teenage pregnancy rates and baseline characteristics including maternal socioeconomic status based on area of residence (Townsend quintiles [30]), were compared using *χ*^2^ tests.

The proportions of pregnancies affected by each adverse outcome were compared initially between women with and without childhood-onset type 1 diabetes. Most outcomes were binary outcomes, modelled using univariable and multivariable logistic regression. Estimates of effects between groups are reported as crude and adjusted ORs (alongside 95% CIs). Hospital admissions during the first year of life were examined using negative binomial regression, with results shown as adjusted incidence rate ratios with 95% CIs. Time to first admission was compared between groups using a Kaplan–Meier curve. Cox regression modelling was used to obtain crude and adjusted HRs. Data were right censored at 1 year follow-up for individuals with no admissions during the first year, time of death or time of moving away from Wales.

We compared the incidence of adverse outcomes between teenage (<20 years) and young adult (≥20 years) mothers with and without childhood-onset type 1 diabetes using the same descriptive and modelling methods outlined above, and including an interaction term (maternal diabetes × teenage mother) in the regression models.

We used HbA_1c_ values from primary care to describe mean HbA_1c_ for teenage and young adult mothers with type 1 diabetes (using the HbA_1c_ closest to conception from the period 1 year prior to conception to the date of delivery). We also described mean HbA_1c_ by trimester of pregnancy for both groups. Unpaired Student’s *t* tests were used to compare HbA_1c_ between groups.

For all statistical analyses, *p* < 0.05 was deemed statistically significant. Data analyses were conducted using IBM SPSS Statistics version 22 (https://www.ibm.com/support/pages/spss-statistics-220-available-download) and Stata version 14 (https://www.stata.com/stata14/).

### Missing data

The proportion of missing data is described in Table 1. Multiple imputation was used to account for missing data under the missing at random assumption [31]. The imputation model included sex, gestational age, birthweight, Townsend quintile, maternal smoking and breastfeeding at 8 weeks, as well as year of birth, maternal age, parity, stillbirths, congenital malformations, pre-eclampsia, delivery by Caesarean section, neonatal death, postneonatal death, maternal type 1 diabetes, multiple births and admissions during the first year of life as predictor variables. HbA_1c_ data were used for exploratory analysis only and were not included in the imputation model. Twenty imputed datasets were generated. The results were consistent with those from complete case analysis. We therefore present the results from analysis of the imputed dataset.

**Table 1:** Baseline characteristics of teenage and young adult mothers with and without childhood-onset type 1 diabetes

Variable	Births to women with childhood-onset type 1 diabetes (*n* = 330)			Births to women without childhood-onset type 1 diabetes (*n* = 197,466)		
	All ages (*n* = 330)	Teenage mothers (*n* = 68, 20.6%)	Young adult mothers (*n* = 262, 79.4%)	All ages (*n* = 197,466)	Teenage mothers (*n* = 43,505, 22.0%)	Young adult mothers (*n* = 153,961, 78.0%)
Maternal characteristics						
Maternal age at delivery (years)	22.8 ± 3.5 (14–32)	17.9 ± 1.2 (14–19)^*^	24.0 ± 2.7 (20–32)^*^	23.0 ± 4.0 (12–33)	17.9 ± 1.1 (12–19)^†^	24.5 ± 3.2 (20–33)^†^
Maternal Townsend quintile						
Quintile 1 (least deprived)	47 (14.7)	9 (13.4)	38 (15.0)	18,380 (9.7)	2567 (6.2)	15,813 (10.7)
Quintile 2	55 (17.2)	10 (14.9)	45 (17.8)	25,627 (13.5)	4396 (10.5)	21,231 (14.3)
Quintile 3	53 (16.6)	12 (17.9)	41 (16.2)	36,256 (19.1)	7308 (17.5)	28,948 (19.5)
Quintile 4	67 (20.9)	17 (25.4)	50 (19.8)	45,312 (23.8)	10,272 (24.6)	35,040 (23.6)
Quintile 5 (most deprived)	98 (30.6)^‡^	19 (28.4)	79 (31.2)	64,496 (33.9)^‡^	17,196 (41.2)^†^	47,300 (31.9)^†^
Missing	10 (3.0)	ND	ND	7395 (3.7)	1766 (4.1)	5629 (3.7)
Maternal smoking during pregnancy	23 (22.1)^‡^	ND	ND	25,437 (32.0)^‡^	7212 (44.9)^†^	18,225 (28.8)^†^
Missing	226 (68.5)	54 (79.4)	172 (65.6)	118,069 (59.8)	27,454 (63.1)	90,615 (58.9)
Duration of maternal diabetes prior to pregnancy (years)	14.3 ± 5.0 (3–30)	9.7 ± 3.6 (3–16)^*^	15.5 ± 4.6 (6–30)^*^	NA	–	–
Age of mother at diagnosis of diabetes (years)	8.0 ± 3.7 (1–14)	7.8 ± 3.4 (1–14)	8.0 ± 3.7 (1–14)	NA	–	–
Pregnancy and birth characteristics						
Gestational age at delivery (weeks)	35.7 ± 3.1 (24–43)^‡^	36.0 ± 2.9 (24–39)	35.7 ± 3.1 (24–43)	39.7 ± 2.2 (24–43)^‡^	39.3 ± 2.3 (24–43)	39.3 ± 2.1 (24–43)
Missing	ND	ND	ND	3438 (1.7)	1452 (3.3)	1986 (1.3)
Birthweight (g)	3233 ± 844 (680–5130)^‡^	3315 ± 791 (1310–4820)	3211 ± 857 (680–5130)	3315 ± 584 (660–5480)^‡^	3252 ± 576 (660–5480)^†^	3333 ± 585 (660–5465)^†^
Missing	ND	ND	ND	437 (0.2)	90 (0.2)	347 (0.2)
Sex of baby						
Male	156 (47.3)	34 (50.0)	122 (46.6)	101,210 (51.3)	22,284 (51.2)	78,926 (51.2)
Female	174 (52.7)	34 (50.0)	140 (53.4)	96,249 (48.7)	21,220 (48.8)	75,029 (48.7)
Missing	0	0	0	7 (0.004)	ND	ND
Delivery by Caesarean section	220 (66.7)^‡^	46 (67.6)	174 (66.4)	36,510 (18.5)^‡^	6241 (14.3)^†^	30,269 (19.7)^†^
Breastfeeding at 8 weeks postpartum	42 (20.7)	ND	ND	31,077 (25.3)	4186 (17.1)^†^	26,891 (27.4)^†^
Missing	127 (38.5)	30 (44.1)	97 (37.0)	74,417 (37.7)	19,014 (43.7)	55,703 (36.2)

Data are mean ± SD (range) or *n* (%). The following baseline characteristics were compared between groups using *χ*^2^ tests: maternal Townsend quintiles, maternal smoking, sex of baby, delivery by Caesarean section and breastfeeding at 8 weeks postpartum. The remaining baseline characteristics (maternal age, duration of maternal diabetes, age of mother at diagnosis of diabetes, gestational age at delivery, birthweight) were compared between groups using unpaired Student's *t* tests

^*^Statistically significant difference (*p* < 0.05) between teenage and young adult mothers with childhood-onset type 1 diabetes

^†^Statistically significant difference (*p* < 0.05) between teenage and young adult mothers without childhood-onset type 1 diabetes

^‡^Statistically significant difference (*p* < 0.05) between women with and without childhood-onset type 1 diabetes

NA, not applicable; ND, not determined (number suppressed as fewer than 5 per cell or would allow a value of less than 5 to be calculated)

### Ethics and information governance

We received approval from the SAIL Information Governance Review Panel. Because the analysis used only anonymised research databases, ethical approval was not required, in line with national ethics committee guidance.

## Results

**Fig. 1:**
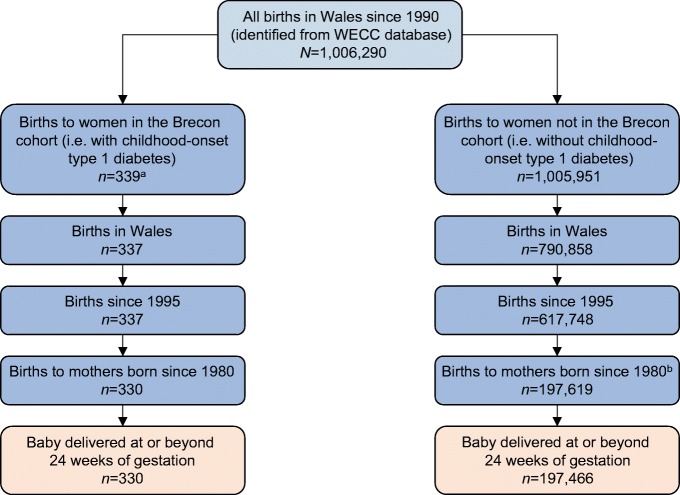
Selection of cohort (individuals excluded because of inaccurate or missing data or failure to meet inclusion criteria for study). ^a^Seven individuals were excluded before this stage because of problems with data linkage (two because of an invalid date of diagnosis, two because of incorrect sex, two because of incorrect date of birth, and one individual that had multiple separate study IDs). ^b^15,889 individuals were excluded at this stage because of missing or invalid maternal date of birth

We identified 197,796 births to girls and women aged 12–33 years eligible for inclusion (Fig. 1), including 330 to girls and women with childhood-onset type 1 diabetes (age 14–32 years).

### Pregnancy rates

Teenagers with diabetes had half the pregnancy rate of teenagers without diabetes (mean annual teenage pregnancy rate between 1999 and 2013: 8.6 vs 18.0 per 1000 teenage girls; *p* < 0.001). In both groups, teenage pregnancy rates were stable over time (see Electronic Supplementary Material [ESM] Table 1). Young adult women with diabetes (20–32 years) had lower pregnancy rates than young adult women without diabetes (20–33 years) (mean annual rate between 1999 and 2013: 49.9 vs 77.3 per 1000 young adult women; *p* < 0.001).

### Comparison between mothers with and without type 1 diabetes

Compared with mothers without type 1 diabetes, those with type 1 diabetes lived in less deprived areas (*p* = 0.005), delivered at earlier gestations (*p* < 0.001) and had higher Caesarean section rates (*p* < 0.001) (Table 1). Smoking rates were lower in mothers with type 1 diabetes (*p* = 0.03), although there were high rates of missing data (Table 1).

**Fig. 2:**
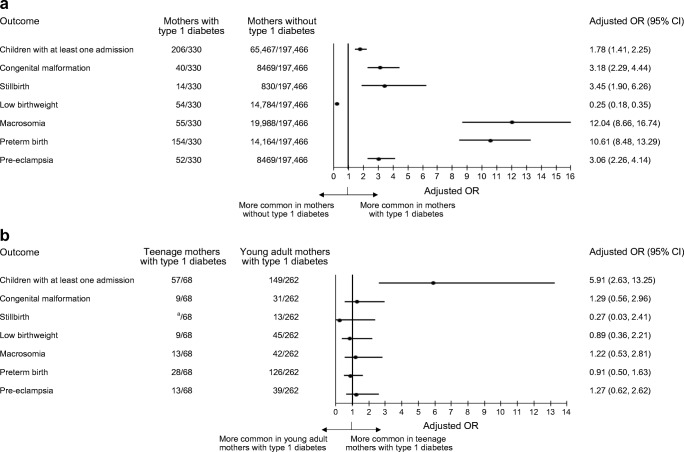
Forest plots showing the adjusted OR (95% CI) for each adverse outcome for babies born to (**a**) women with vs without childhood-onset type 1 diabetes; and (**b**) teenage vs young adult mothers with childhood-onset type 1 diabetes. ^a^Number suppressed as less than five

All adverse outcomes were more common in mothers with diabetes than mothers without diabetes (ESM Table 2). After adjusting for confounders, maternal diabetes was associated with an increased risk of all adverse outcomes, except low birthweight (Fig. 2a). After adjusting for gestational age (which was significantly lower for babies of women with type 1 diabetes), the odds of low birthweight were higher for babies of women without diabetes (ESM Table 2, Fig. 2a).

The children of women with diabetes had more admissions during the first year of life (median 3.00 [interquartile range 2.00–4.25] vs 1.00 [1.00–2.00]; adjusted incidence rate ratio 1.41 [95% CI 1.22, 1.65]). Time to first admission was also shorter for the children of women with diabetes (adjusted HR 1.62 [95% CI 1.41, 1.86]). The most common reason for admission was perinatal complications for the children of women with diabetes (41.4% of admissions) and respiratory infections for the children of women without diabetes (24.1%).

After discounting admissions occurring during the first 28 days of life, there was no longer a significant difference in the proportion of children admitted to hospital during the first year between women with and without diabetes (adjusted OR 1.02 [95% CI 0.80, 1.29]), suggesting the excess was due to perinatal complications. The most common perinatal complications among babies of women with diabetes were the consequences of complications of pregnancy, labour and delivery (34.8%), followed by neonatal hypoglycaemia (32.0%; ESM Table 3).

### Comparison between teenage and young adult mothers with childhood-onset type 1 diabetes

Our study included 68 teenage mothers (age 14–19 years, mean 17.9 years) and 262 young adult mothers (age 20–32 years, mean 24.0 years) with childhood-onset type 1 diabetes. The mean duration of diabetes was 9.7 years for teenage mothers and 15.5 years for young adult mothers. There was no significant difference in socioeconomic status between teenage and young adult mothers with diabetes (*p* = 0.85) (Table 2). This is in contrast to those without diabetes, among whom the teenagers lived in more deprived areas than the young adults (*p* < 0.001; Table 2).

**Table 2:** Socioeconomic status of teenage and young adult mothers with and without childhood-onset type 1 diabetes

Maternal Townsend quintile	All ages (*n* = 197,796)	Teenage mothers (*n* = 43,573)	Young adult mothers (*n* = 154,223)	Significance of comparison between teenage and young adult mothers
Mothers with childhood-onset type 1 diabetes, *n*	*n* = 330	*n* = 68	*n* = 262	*p* = 0.85
Quintile 1 (least deprived)	47 (14.7)	9 (13.4)	38 (15.0)	
Quintile 2	55 (17.2)	10 (14.9)	45 (17.8)	
Quintile 3	53 (16.6)	12 (17.9)	41 (16.2)	
Quintile 4	67 (20.9)	17 (25.4)	50 (19.8)	
Quintile 5 (most deprived)	98 (30.6)	19 (28.4)	79 (31.2)	
Missing	10 (3.0)	ND	ND	
Mothers without childhood-onset type 1 diabetes, *n*	*n* = 197,466	*n* = 43,505	*n* = 153,961	*p* <0.001
Quintile 1 (least deprived)	18,380 (9.7)	2567 (6.2)	15,813 (10.7)	
Quintile 2	25,627 (13.5)	4396 (10.5)	21,231 (14.3)	
Quintile 3	36,256 (19.1)	7308 (17.5)	28,948 (19.5)	
Quintile 4	45,312 (23.8)	10,272 (24.6)	35,040 (23.6)	
Quintile 5 (most deprived)	64,496 (33.9)	17,196 (41.2)	47,300 (31.9)	
Missing	7395 (3.7)	1766 (4.1)	5629 (3.7)	
Significance of comparison between mothers with and without childhood-onset type 1 values	*p* = 0.005	*p* = 0.046	*p* = 0.046	

Data are *n* (%)

Comparisons between groups were made using *χ*^2^ testing and the resulting *p* values are shown

ND, not determined (number suppressed as fewer than 5 per cell or would allow a value of less than 5 to be calculated)

Whereas pregnancy outcomes were worse in teenagers with diabetes than teenagers without diabetes (ESM Table 4), outcomes were not significantly different between teenage and young adult mothers with diabetes (ESM Table 4, Fig. 2b), except for a higher admission rate during the first year of life for babies of teenage mothers with diabetes (adjusted OR 5.91 [95% CI 2.63, 13.25]). The effect of maternal diabetes on time to first admission was stronger in teenage mothers (adjusted HR 3.01 [95% CI 2.31, 3.91]) than in young adult mothers (adjusted HR 1.42 [95% CI 1.20, 1.60]), and the interaction (maternal diabetes by teenage mother) was significant (adjusted HR 2.00 [95% CI 1.47, 2.71], *p* < 0.01). The most common reason for admission in the children of both teenage and young adult mothers with diabetes was perinatal complications. Even after discounting admissions occurring in the first 28 days of life, the babies of teenage mothers with diabetes remained at higher risk of admission than those of young adult mothers with diabetes (adjusted OR 2.11 [95% CI 1.20, 3.71], *p* = 0.01). After the first 28 days of life, the most common reason for admission in the children of both teenage and young adult mothers with diabetes was respiratory infections (30.6% and 24.7%, respectively).

### Glycaemic control

HbA_1c_ values were available for 208 (63.0%) mothers with diabetes (61.8% teenage mothers and 63.4% young adult mothers) from 1 year prior to conception to the date of delivery. The ages of those with and without HbA_1c_ readings were comparable (mean 22.8 vs 22.6 years, *p* = 0.64), and there was no significant difference in the socioeconomic background of those with and without HbA_1c_ readings (*p* = 0.65).

Glycaemic control was poor in teenage and young adult mothers with diabetes (overall mean HbA_1c_ using value closest to conception from values taken from 1 year prior to conception to delivery date 80.5 mmol/mol [9.5%]). There was no significant difference in mean HbA_1c_ between teenage and young adult mothers (81.3 vs 80.2 mmol/mol, [9.6% vs 9.5%] respectively, *p* = 0.78; Table 3).

**Table 3:** Glycaemic control by stage of pregnancy in mothers with type 1 diabetes

Timing of readings	Mothers, all ages (*n* = 330)			Teenage mothers (*n* = 68)			Young adult mothers (*n* = 262)			
	With valid readings^a^	HbA_1c_ (mmol/mol)	HbA_1c_ (%)	With valid readings^a^	HbA_1c_ (mmol/mol)	HbA_1c_ (%)	With valid readings	HbA_1c_ (mmol/mol)	HbA_1c_ (%)	*p* value
Closest value to conception^b^	208 (63.0)	80.5 (35.5–138.2)	9.5 (5.4–14.8)	42 (61.8)	81.3 (53.0–125.1)	9.6 (7.0–13.6)	166 (63.4)	80.2 (35.5–138.2)	9.5 (5.4–14.8)	0.78
1 month prior to conception to end of first trimester^c^	101 (30.6)	79.0 (43.0–136.0)	9.4 (6.1–14.6)	17 (25.0)	85.5 (54.0–116.3)	10.0 (7.1–12.8)	84 (32.1)	77.6 (43.0–136.0)	9.3 (6.1–14.6)	0.12
During the second trimester^d^	76 (23.0)	61.2 (38.7–101.0)	7.7 (5.7–11.4)	17 (25.0)	67.5 (39.8–101.0)	8.3 (5.8–11.4)	59 (22.5)	59.4 (38.7–90.1)	7.6 (5.7–10.4)	0.05
During the third trimester^e^	55 (16.7)	56.5 (35.5–122.9)	7.3 (5.4–13.4)	10 (14.7)	67.4 (40.9–122.9)	8.3 (5.9–13.4)	45 (17.2)	54.0 (35.5–88.0)	7.1 (5.4–10.2)	0.01

Data are *n* (%) (mothers with valid readings) and mean (range) HbA_1c_

*p* values are shown for comparisons between teenage and young adult mothers as derived from unpaired Student's *t* tests

^a^The mean ages of those with and without at least one valid HbA_1c_ reading during the period from 1 year prior to conception to the date of delivery were comparable (mean 22.8 vs 22.6 years, *p* = 0.64), and there was no significant difference in the socioeconomic background of those with and without HbA_1c_ readings (*p* = 0.65). The mean ages of those with and without HbA_1c_ readings during each trimester were also comparable (first trimester: 23.0 vs 22.6 years, *p* = 0.41; second trimester: 22.5 vs 22.8 years, *p* = 0.48; third trimester: 22.3 vs 22.8 years, *p* = 0.29) and there was no significant difference in the socioeconomic background of those with and without HbA_1c_ readings (first trimester, *p* = 0.52; second trimester, *p* = 0.92; third trimester, *p* = 0.29)

^b^From readings taken between 1 year prior to conception and date of delivery

^c^Defined as the end of the 12th week of pregnancy

^d^Defined as between the beginning of the 13th week of pregnancy and the end of the 27th week of pregnancy

^e^Defined as from the beginning of the 28th week of pregnancy until the date of delivery

HbA_1c_ data were analysed by trimester of pregnancy, but these data should be interpreted cautiously because of a significant proportion of missing data (Table 3). Glycaemic control improved with each trimester of pregnancy in both teenage and young adult mothers, but improved to a greater degree in young adult mothers (Table 3). Whereas there was no statistically significant difference in glycaemic control between teenage and young adult mothers during the first trimester, in the second trimester young adult mothers had borderline better glycaemic control than teenage mothers (mean HbA_1c_ 59.4 vs 67.5 mmol/mol [7.6% vs 8.3%], respectively, *p* = 0.05). By the third trimester, glycaemic control was significantly better in young adult mothers (54.0 vs 67.4 mmol/mol [7.1% vs 8.3%], respectively, *p* = 0.01) (Table 3).

## Discussion

This study represents a large, community-based study comparing pregnancy characteristics and outcomes between teenage mothers with childhood-onset type 1 diabetes (14–19 years) and young adult women with childhood-onset type 1 diabetes (20–32 years). In our study, the proportion of teenage girls with diabetes with a pregnancy resulting in legal birth was half that of teenage girls without diabetes. Teenage pregnancy in girls and women with diabetes was not associated with social deprivation, in contrast to teenage pregnancy in those without diabetes. Obstetric outcomes were poor in teenagers with diabetes but not worse than in young adult mothers with diabetes, likely reflecting poor glycaemic control in both groups. The infants of teenagers with type 1 diabetes had an excess of hospital admissions during the first year of life.

Little is known about the frequency of teenage pregnancy in girls and women with type 1 diabetes. In the general population, although teenage pregnancy rates across Europe are falling, they remain high in many countries [32, 33]. We report a mean annual rate of teenage pregnancies resulting in legal births of 18 per 1000 teenage girls for teenagers without diabetes during the period 1999–2013. This is comparable with data published regarding teenage pregnancy in Wales by the Office for National Statistics for England and Wales [32]. Teenage pregnancy rates are lower in other European countries, (e.g. rates for teenage pregnancies resulting in birth in 2011 were 5 per 1000 girls and women aged 15–19 years in Denmark, 6 per 1000 in Sweden and 10 per 1000 in Spain) but higher in the USA (34 per 1000) and New Zealand (26 per 1000) [33].

In our study, teenagers with type 1 diabetes had half the pregnancy rate resulting in legal birth seen in teenagers without diabetes. This finding has not been previously documented and the reasons behind it are unclear, but may represent the influence of parents/carers or healthcare professionals.

Teenage pregnancy is reportedly more common in deprived communities [11, 34–36]. We replicated this observation in women without type 1 diabetes. However, in our study, teenage pregnancy in the context of diabetes was not associated with higher levels of deprivation than in young adults with diabetes. Since our study contained a relatively small number of teenagers with diabetes, larger studies are required to validate this finding.

In our cohort, glycaemic control around the point of conception was poor among teenagers, as reported elsewhere for non-pregnant teenagers [14, 15]. Glycaemic control was equally poor among young adult women. As such, the mean HbA_1c_ for all women with type 1 diabetes in our cohort was relatively high, far from targets set by the National Institute for Health and Care Excellence [37] and worse than reported in the UK National Pregnancy in Diabetes Audit [38]. This may reflect the relatively young age of our cohort (mean age 22.8 years vs a median age of 30.0 years in the 2016 National Audit) [38]. Data from the Type 1 Diabetes Exchange demonstrate that glycaemic control is typically poorest in teenagers, with significant improvements tending not to occur until a person’s late 20s [14]. Data from the National Pregnancy in Diabetes Audit demonstrate that women who achieve HbA_1c_ targets during pregnancy are typically older and live in less deprived regions [39]. Therefore, the relatively young age of our ‘young adult mothers’, as well as the fact that our cohort represents a moderately deprived community, likely account for our high mean HbA_1c_ levels.

Data from the National Audit demonstrate that glycaemic control typically improves as pregnancy progresses [38]. Similarly, in our cohort, glycaemic control improved with each trimester of pregnancy. The improvement in glycaemic control was more significant in young adult mothers, and by the third trimester young adult mothers had significantly better glycaemic control than teenage mothers. While this might suggest that interventions to improve glycaemic control during pregnancy were less successful in teenagers in our cohort, the data should be interpreted cautiously due to a high proportion of missing data. Missing data likely reflects a combination of not all general practices in Wales being registered with SAIL and poor recording of HbA_1c_ during pregnancy in primary care in Wales. Further studies are required to provide a more comprehensive description of the relationship between maternal age, changes in HbA_1c_ during pregnancy and pregnancy outcomes.

Duration of diabetes is likely to confound the relationship between maternal age and pregnancy outcomes. Older women may have a longer duration of diabetes and more microvascular complications, such as nephropathy, which is particularly associated with poor pregnancy outcomes [40, 41]. However, in our cohort, exploratory analyses revealed no significant difference in glycaemic control or pregnancy outcomes between women with a duration of diabetes of less than 10 years and those with a longer duration (ESM Table 5). Data regarding signs of nephropathy were limited, with only 59 (17.9%) mothers with diabetes having a urine albumin/creatinine ratio available for the year prior to conception; of these, 14 had a result >3 mg/mmol.

A recent report from an insurance-based cohort of 119 million people in the USA described pregnancy outcomes in 639 teenagers with pregestational diabetes, and confirmed increased pregnancy complication rates compared with teenagers without diabetes [16]. Consistent with our findings, the researchers reported an increased risk of pre-eclampsia, preterm delivery and high birthweight [16]. However, they did not differentiate between types of pregestational diabetes and no socioeconomic data were included. Furthermore, this study did not compare pregnancy outcomes between teenagers and older women with diabetes.

Our cohort includes three times more teenage mothers with type 1 diabetes than the largest previously published study comparing pregnancy outcomes between teenagers and older women with type 1 diabetes [17]. Our findings differ from those of Carmody et al, who observed worse glycaemic control during pregnancy in teenagers than older mothers and an increased rate of congenital malformations [17]. However, their study included fewer teenagers (*n* = 18) and compared them with all mothers with type 1 diabetes, whereas our cohort was censored at the age of 35 years, resulting in a lower mean age for ‘older mothers’ (24.0 vs 31.0 years). It is possible that the relatively good outcomes for older mothers in Carmody et al’s cohort was influenced by their better glycaemic control [17]. The researchers did not report socioeconomic data or rates of teenage pregnancy in type 1 diabetes.

We report an excess of hospital admissions during the first year of life among babies of teenage mothers with type 1 diabetes. It is recognised that, in the general population, babies born to teenage mothers have more hospital admissions, partly because of socioeconomic factors [42]. However, our data show that maternal type 1 diabetes has an additive effect, conferring an increased risk of admission during the first year of life beyond that observed among the offspring of teenage mothers in the background population. Since this remained significant even after discounting admissions in the first 28 days of life, the excess of admissions cannot be entirely explained by perinatal complications. Increased morbidity after the perinatal period or differences in healthcare-seeking behaviour may be contributing factors.

It is clear that pregnancy and early infant outcomes remain poor among teenage and young adult women with type 1 diabetes in Wales, and poor glycaemic control is likely the main reason for this. To improve outcomes, a multifaceted approach combining education with the latest advances in pharmacology and technology is required. Ensuring that young women with diabetes have access to effective contraception is essential. In addition, it is important that young women can access advice regarding sexual health and pregnancy, and that early referral to specialist services occurs as soon as pregnancy is reported. Newer technologies, such as continuous glucose monitoring, may help to address uncertainty about adjusting insulin dosage in the face of changing insulin requirements during pregnancy, and translate to better obstetric outcomes [43]. Finally, in order to reduce the excess of admissions seen among the infants of teenage mothers with diabetes, support for young mothers with diabetes should continue beyond delivery.

The main limitation of this study is the relatively small number of teenage pregnancies in girls and women with type 1 diabetes. In addition, since the Brecon cohort includes only individuals diagnosed with type 1 diabetes prior to age 15 years, the background population includes individuals with type 1 diabetes diagnosed after age 15 years and with type 2 diabetes. However, these individuals are unlikely to account for a large number of women in the background population, particularly given the age distribution of our cohort. Since our cohort contains only pregnant women up to the age of 35 years, and contains more women at the younger age of the spectrum, we have not described pregnancy outcomes in women at the older end of the reproductive-age spectrum, in whom glycaemic control and therefore pregnancy outcomes may be better. Further studies are required, containing larger numbers of teenage and older mothers, to advance our understanding of the relationship between maternal age and pregnancy outcomes in type 1 diabetes. Our cohort included only pregnancies resulting in legal births, as pregnancies resulting in miscarriage or termination are not recorded within the SAIL Databank. Therefore, while our data provide an accurate representation of pregnancies resulting in legal births, further work is required to explore whether the proportion of pregnancies conceived is similar between teenagers with and without type 1 diabetes. Finally, a high proportion of missing data for HbA_1c_ and urine albumin/creatinine ratio results within the primary care dataset limited our ability to incorporate these into our regression model.

### Conclusion

Teenage girls with childhood-onset type 1 diabetes in Wales are less likely to have children than teenage girls without diabetes. Pregnancy in teenagers with type 1 diabetes, unlike in the background population, is not associated with social deprivation. Among girls and women with type 1 diabetes, HbA_1c_ during and before pregnancy was not well documented and, when reported, glycaemic control was poor in teenage and young adult mothers. Pregnancy outcomes were comparable between teenage and young adult mothers with type 1 diabetes, but hospital admissions during the first year of life were five times more common among babies of teenage mothers with type 1 diabetes than those of young adult mothers with diabetes.

## Electronic supplementary material

ESM (PDF 136 kb)

## Data Availability

Data from the following datasets were used: The Brecon Register, WECC, CARIS, Patient Episod Database for Wales (PEDW) and Primary care GP dataset. These pseudonymised, population-based datasets were linked in the SAIL Databank to create an e-cohort of legal births to women under 35 years between 1995 and 2013 in Wales. Researchers did not have access to personal identifiable data. Access to data held within the SAIL Databank is dependent on making a formal application to SAIL and gaining approval from the SAIL Information Governance Review Panel.

## Abbreviations

CARIS: Congenital Anomaly Register and Information Service
SAIL: Secure Anonymised Information Linkage
WECC: Wales Electronic Cohort for Children
