# Supplementary material for: Pregnancy in teenagers diagnosed with type 1 diabetes mellitus in childhood: a national population-based e-cohort study
Source: Diabetologia. 2019 Dec 20;63(4):799–810. doi: 10.1007/s00125-019-05063-w (PMC7054376; doi:10.1007/s00125-019-05063-w)
Supplement: Supplementary file 1 — (PDF 136 kb) [file 125_2019_5063_MOESM1_ESM.pdf]

Electronic Supplementary Material (ESM)

ESM Table 1: Teenage pregnancy rates

	Teenage girls with type 1 diabetes experiencing pregnancy resulting in legal birth	Teenage girls without type 1 diabetes experiencing pregnancy resulting in legal birth
1999-2003[a]	17 (7.3 per 1000 95% CI 4.6, 11.7)	14,526 (19.2 per 1000, 95% CI 18.9, 19.5)
2004-2008	26 (9.6 per 1000, 95% CI 7.6, 16.2)	15,043 (19.4 per 1000, 95% CI 19.1, 19.7)
2009-2013	25 (8.8 per 1000, 95% CI 7.3, 15.8)	11,206 (15.3 per 1000, 95% CI 15.0, 15.6)
1999-2013	68 (8.6 per 1000 95% CI 6.8, 10.9)	40,775 (18.0 per 1000, 95% CI 17.8, 18.2)

Values are absolute numbers of teenage pregnancies (mean annual rate of teenage pregnancies per 1000 teenage girls with 95% confidence intervals for the specified time period)

[a] Since there were no births to teenage girls with type 1 diabetes prior to 1999 in our cohort, comparison of teenage pregnancy rates begins in 1999.

It is not possible to report annual teenage pregnancy rates due to the small numbers of teenage pregnancies in women with type 1 diabetes, and the risk of compromising the anonymity of these individuals.

ESM table 2: Adverse pregnancy outcomes in women with and without childhood-onset type 1 diabetes

Adverse outcome	Women with childhood-onset type 1 diabetes n=330	Women without childhood-onset type 1 diabetes n=197,466	Crude odds ratio (95% CI)	Adjusted odds ratio (95% CI)
Pre-eclampsia	52 (15.8%)	10,557 (5.4%)	3.31 (2.46, 4.46)	3.06 (2.26, 4.14) [a]
Preterm births (births <37th week of pregnancy)	154 (47.1%)	14,164 (7.3%)	11.36 (9.14, 14.12)	10.61 (8.48, 13.29) [b]
Macrosomia (birth weight ≥ 4000 grams)	55 (16.9%)	19,988 (10.1%)	1.77 (1.53, 2.06)	12.04 (8.66, 16.74) [c]
Low birth weight (birth weight ≤2500 grams)	54 (16.6%)	14,784 (7.5%)	2.58 (2.23, 2.98)	0.25 (0.18, 0.35) [c]
Stillbirths	14 (4.2%)	830 (0.42%)	10.50 (6.12, 18.01)	3.45 (1.90, 6.26) [d]
Congenital malformations	40 (12.1%)	8469 (4.3%)	3.08 (2.21, 4.29)	3.18 (2.29, 4.44) [a]
Children with at least 1 hospital admission during first year of life	206 (62.4%)	65,467 (33.2%)	3.35 (2.68, 4.19)	1.78 (1.41, 2.25) [e]

Number (%) of births affected by each adverse outcome are shown, alongside crude and adjusted odds ratios for adverse outcomes in women with childhood-onset type 1 diabetes compared with women without childhood-onset type 1 diabetes (reference group), as derived from logistic regression modelling.

[a] Adjusted models control for maternal age, maternal deprivation quintile, parity, maternal smoking, and sex of baby
[b] Additionally adjusted for pre-eclampsia, and congenital malformation
[c] Additionally adjusted for pre-eclampsia, congenital malformation, and gestational age at delivery
[d] Additionally adjusted for gestational age at delivery, and birth weight
[e] Additionally adjusted for delivery by Caesarean section, gestational age at delivery, birth weight, breastfeeding, and congenital malformation

ESM Table 3: Causes of perinatal admissions amongst the infants of mothers with childhood-onset type 1 diabetes.

Cause of perinatal admission	Number of admissions (% of all perinatal admissions)
Fetus and newborn affected by maternal factors and by complications of pregnancy, labour and delivery, and disorders related to length of gestation, fetal growth, and birth trauma	62 (34.8%)
Transitory endocrine and metabolic disorders specific to fetus and newborn[a]	57 (32.0%)
Respiratory and cardiovascular disorders specific to the perinatal period	21 (11.8%)
Haemorrhagic and haematological disorders of fetus and newborn	13 (7.3%)
Digestive system disorders of fetus and newborn	7 (3.9%)
Other	18 (10.1%)

The number of admissions is shown alongside the % of all perinatal admissions (n=178.) Causes of perinatal admissions are categorised and defined according to the International Classification of Diseases, 10th revision [30].

[a] all admissions coded as being for transitory endocrine and metabolic disorders specific to fetus and newborn were due to neonatal hypoglycaemia.

It is not possible to provide this data for teenage and young adult women with type 1 diabetes separately due to the low numbers of infants born to teenage mothers with type 1 diabetes being admitted for these individual reasons, and the risk of compromising the anonymity of individuals as a result.

ESM table 4: Adverse pregnancy outcomes in teenage and young adult mothers with and without childhood-onset type 1 diabetes

	Women with childhood-onset type 1 diabetes n=330			Women without childhood-onset type 1 diabetes n=197,466			
	Teenage mothers n=68	Young adult mothers n=262	Adjusted odds ratio	Teenage mothers n=43,506	Young adult mothers n=153,960	Adjusted odds ratio	Adjusted odds ratio for interaction term maternal diabetes x teenage mother
Pre-eclampsia	13 (19.1%)	39 (14.9%)	1.27 (0.62, 2.62)[a]	2513 (5.8%)	8044 (5.2%)	0.93 (0.89, 0.98)[a]	1.26 (0.62, 2.54) [a]
Preterm births (births <37th week of pregnancy)	28 (41.2%)	126 (48.1%)	0.91 (0.50, 1.63)[b]	3412 (8.1%)	10,752 (7.1%)	1.08 (1.03, 1.13)[b]	0.69 (0.40, 1.21) [b]
Macrosomia (birth weight ≥ 4000 grams)	13 (19.1%)	42 (16.0%)	1.22 (0.53, 2.81)[c]	3335 (7.7%)	16,653 (10.8%)	0.90 (0.87, 0.94)[c]	1.48 (0.70, 3.16) [c]
Low birth weight (birth weight ≤2500 grams)	9 (13.2%)	45 (17.2%)	0.89 (0.36, 2.21)[c]	3691 (9.1%)	11,093 (7.2%)	0.91 (0.86, 0.96)[c]	0.83 (0.36, 1.92) [c]
Stillbirths	ND	ND	0.27 (0.03, 2.41)[d]	156 0.4%)	674 (0.4%)	0.68 (0.56, 0.82)[d]	0.43 (0.05, 3.74) [d]
Congenital malformations	9 (13.2%)	31 (11.8%)	1.29 (0.56, 2.96)[a]	2015 (4.6%)	6454 (4.2%)	1.05 (1.00, 1.11)[a]	1.04 (0.47, 2.31) [a]
Children with at least 1 hospital admission during first year of life	57 (83.8%)	149 (56.9%)	5.91 (2.63, 13.25)[e]	16,354 (37.6%)	49,113 (31.9%)	1.22 (1.19, 1.25)[e]	3.54 (1.74, 7.18) [e]

Number (%) of births affected by each adverse outcome by group are shown. Comparison was made between births to teenage and young adult mothers (reference group) for women with and without childhood-onset type 1 diabetes, using logistic regression modelling, with results shown as crude and adjusted odds ratios. An interaction term (maternal diabetes x teenage mother) was included in the regression model.

[a] Adjusted for maternal deprivation quintile, parity, maternal smoking, and sex of baby
[b] Additionally adjusted for pre-eclampsia, congenital malformations
[c] Additionally adjusted for pre-eclampsia, congenital malformations, and gestational age at delivery
[d] Additionally adjusted for gestational age at delivery, and birth weight
[e] Additionally adjusted for delivery by Caesarean section, gestational age at delivery, birth weight, breastfeeding, and congenital malformations

ND, not determined (number suppressed as fewer than five per cell or would allow a value of less than five to be calculated)

ESM Table 5: Subgroup analysis comparing pregnancy outcomes for women with childhood-onset type 1 diabetes of >10 years' duration, and women with childhood-onset type 1 diabetes of ≤10 years' duration.

	Mothers with type 1 diabetes >10 years' duration (n=246)	Mothers with type 1 diabetes ≤10 years' duration (n=84)	Crude odds ratio	Adjusted odds ratio
Pre-eclampsia	37 (15.0%)	15 (17.9%)	1.23 (0.64, 2.37)	1.02 (0.46, 2.24)[a]
Preterm births (births <37th week of pregnancy)	121 (49.4%)	33 (40.2%)	0.88 (0.47, 1.66)	0.81 (0.48, 1.38) [b]
Macrosomia (birth weight ≥ 4000grams)	34 (14.0%)	21 (24.3%)	2.08 (1.13, 3.83)	2.24 (0.96, 5.21) [c]
Low birth weight (birth weight ≤2500 grams)	42 (17.3%)	12 (14.5%)	0.82 (0.42, 1.60)	1.12 (0.39, 3.17) [c]
Stillbirth	ND	ND	0.79 (0.22, 2.91)	1.11 (0.23, 5.32)[d]
Congenital malformations	30 (12.2%)	10 (11.9%)	0.97 (0.45, 2.09)	0.91 (0.37 2.26) [a]
Children with at least 1 hospital admission during first year of life	148 (60.2%)	58 (69.0%)	1.48 (0.87, 2.51)	0.70 (0.34, 1.45)[e]

Number (%) of births affected by each adverse outcome are shown, alongside crude and adjusted odds ratios for adverse outcomes in women with childhood-onset type 1 diabetes of >10 years' duration compared with women with childhood-onset type 1 diabetes of ≤10 years' duration (reference group), as derived from logistic regression modelling.

[a] Adjusted for maternal age, maternal deprivation quintile, parity, maternal smoking, and sex of baby
[b] Additionally adjusted for pre-eclampsia, congenital malformations
[c] Additionally adjusted for pre-eclampsia, congenital malformations, and gestational age at delivery
[d] Additionally adjusted for gestational age at delivery, and birth weight
[e] Additionally adjusted for delivery by Caesarean section, gestational age at delivery, birth weight, breastfeeding, and congenital malformations

ND, not determined (number suppressed as fewer than five per cell or would allow a value of less than five to be calculated)
